# Between scientific capital and emotional capital: a cross-sectional study on the empathetic clinical gaze in Swedish medical education

**DOI:** 10.3389/fmed.2026.1876057

**Published:** 2026-08-27

**Authors:** Lena Halawi, Atika Khalaf

**Affiliations:** 1 Faculty of Health Science, Kristianstad University, Kristianstad, Sweden; 2 Hind Bint Maktoum College of Nursing and Midwifery, Mohammed Bin Rashid University of Medicine and Health Sciences, Dubai Health, Dubai, United Arab Emirates

**Keywords:** clinical detachment, clinical empathy, emotional capital, emotional engagement, medical education, patient-provider relationship

## Abstract

**Introduction:**

Clinical empathy was examined among Swedish medical students to understand how empathic engagement evolves during training. The study explored both quantitative patterns and qualitative reflections related to the Empathetic Clinical Gaze (ECG).

**Methods:**

Clinical empathy was examined using a cross-sectional quantitative survey of 251 Swedish medical students with the Empathetic Clinical Gaze scale (ECG scale). To contextualize the quantitative findings, a secondary qualitative content analysis of brief to moderately reflective written comments from open-ended item evaluation prompts was carried out.

**Results:**

Quantitatively, no significant gender or age differences in ECG were observed (*p* > 0.05), while early-year students demonstrated higher ECG levels than those in later-years (39.1% vs. 25.5%; *p* = 0.030). Qualitatively, students described a growing tension between emotional resonance and clinical pragmatism. Early-year students emphasized affective engagement, whereas later-year students reported a shift toward cognitive empathy to manage diagnostic efficiency, workload pressures, and risks of emotional exhaustion. Two themes emerged: (1) Clinical detachment- The practice of applying empathy in a clinical context and (2) The application and significance of empathy in patient encounters. These themes reflect institutional and environmental influences that mirror global challenges in sustaining an empathetic clinical gaze during clinical training. Across groups, students described empathy as a context-dependent skill shaped by workload, time constraints, and professional norms.

**Discussion:**

The study highlights the need for curricular strategies that support a sustainable empathetic clinical gaze, including longitudinal training, reflective practice, and scenario-based role-play. These findings contribute to international efforts to reconceptualize empathy as a dynamic, context-sensitive competency and offer actionable insights for educators seeking to address challenges in sustaining clinical empathy.

## Introduction

Clinical empathy is widely recognized as a core component of medical professionalism, supporting effective communication, strengthening patient-clinician relationships, and contributing to improved clinical outcomes (1, 2). However, despite its importance, clinical empathy remains conceptually challenged within medical education, where it is often divided into cognitive and affective dimensions. However, the dichotomy between cognitive and affective empathy has contributed to an overemphasis on cognitive empathy, while downplaying affective empathy. Scholars such as Halpern and Shapiro have argued that this separation oversimplifies the complexity of clinical empathy and risks promoting “detached concern,” a stance that aligns with Foucault's notion of the scientific medical gaze, in which biomedical rationality and objectivity overshadow patients' lived experiences (3–8). This highlights the importance of clearly articulating what distinguishes cognitive from affective empathy, and how each contributes to clinical communication.

Cognitive empathy involves perspective-taking and the ability to intellectually understand a patient's situation, for example, acknowledging a patient's worry by saying, “*I can see why this would be concerning; let's look at what it means together.”* This allows clinicians to interpret and validate the patient's concerns without becoming emotionally overwhelmed. Affective empathy, in contrast, is about the clinician's emotional attunement and ability to be moved by the patient's experience in a regulated and professional manner. This does not involve sharing the patient's suffering, but rather conveying warmth, presence, and compassion through tone of voice, facial expression, and body language. For instance, a clinician might soften their tone, adjust their posture to show attentiveness, or gently say, “*I can see this is difficult, and I'm here with you,”* to convey warmth and emotional presence. According to Halpern and Shapiro (4, 5), emotions cannot be separated from cognition, and emotional understanding is essential for effective communication. Crucially, emotional attunement enables this understanding by allowing clinicians to perceive and respond to the emotional nuances of the clinical encounter. Together, these dimensions form a relational process in which clinicians both understand and connect with patients, fostering trust, security, and person-centered communication. However, medical education further reinforces the tension between these dimensions. While curriculums frequently emphasize clinical empathy and compassion, the hidden curriculum is said to often promote emotional restraint, efficiency, and clinical detachment. In this regard, the hidden curriculum refers to the implicit norms, values, and expectations that students learn through the culture and everyday practices of medical training rather than through formal teaching (5). These unspoken lessons, such as prioritizing efficiency, emotional restraint, or clinical detachment, are conveyed through role-modeling, institutional routines, and the social organization of clinical work. As a result, the hidden curriculum can shape how medical students understand and express clinical empathy, sometimes reinforcing behaviors that conflict with the formally taught ideals of compassion and person-centered care. As Hafferty and others have noted, medical schools may implicitly encourage “*an ethic of detachment, self-interest, and objectivity,”* shaping students' outlooks toward patients and influencing how empathy is learned, practiced, and valued (5). Research on empathy trajectories among medical students reflects these pressures, with some studies reporting declines during clinical training (9–14) and others showing stability or improvement depending on educational context (15, 16). Gender differences have also been commonly observed, with female students often scoring higher on empathy measures (15, 16). Furthermore, recent mixed-methods studies have highlighted the value of combining quantitative empathy measures with qualitative exploration of students' experiences. For example, communication-skills training interventions have demonstrated improvements in empathy scores while simultaneously revealing how students conceptualize cognitive, behavioral, and moral dimensions of empathy in clinical encounters (17).

Mixed-methods educational research has further demonstrated that empathy development is influenced by reflective and experiential learning opportunities. For instance, qualitative findings from concurrent mixed-methods interventions have shown that active listening, emotional expression, and self-reflection contribute to students' understanding of empathic practice beyond what quantitative scores alone can reveal (18). Emerging mixed-methods evidence also suggests that immersive educational strategies, including virtual reality simulations, may enhance empathy by helping students engage with patients' lived experiences while simultaneously providing qualitative insights into how empathy is perceived and enacted (19).

Although several validated instruments assess empathy in clinical contexts, such as the Jefferson Scale of Empathy (JSE), the CARE Measure, and the Therapist Empathy Scale (TES), these tools primarily conceptualize empathy as a cognitive construct (20). As a result, they may underrepresent the emotional and relational dimensions central to clinical encounters. The JSE, for example, emphasizes understanding, perspective-taking, and compassionate care, but conceptualizes empathy predominantly as a cognitive trait (20, 21). Yet emotional attunement is crucial for facilitating genuine understanding and communication and cannot be separated from cognition in clinical practice (6–8).

To address this limitation, Halawi et al. (22) developed and validated the Empathetic Clinical Gaze Scale (ECGS), a self-report instrument grounded in Halpern's concept of “engaged curiosity,” which conceptualizes clinical empathy as an empathetic clinical gaze i.e., a unified integration of cognitive understanding and emotional attunement. The ECGS was developed to capture clinicians' genuine interest in patients' emotional experiences and to assess empathy as a holistic perceptual stance rather than a set of discrete components. Additionally, the study focused on the psychometric development and initial validation of the ECGS among Swedish medical students.

The ECGS is explicitly grounded in the Empathetic Clinical Gaze (ECG) framework, which conceptualizes clinical empathy as a unified perceptual stance integrating cognitive understanding with emotional attunement through engaged curiosity (22). This represents a distinct conceptual refinement rather than a re-labeling of constructs such as clinical empathy, emotional attunement, or compassion. Whereas, traditional frameworks separate cognitive and affective components or focus on isolated capacities, such as perspective-taking, emotional resonance, or relational behaviors, ECG specifies the underlying curiosity-driven perceptual orientation that precedes and shapes these actions. It operationalizes how clinicians attend to subtle verbal and non-verbal cues to explore the deeper meaning of a patient's distress, rather than merely acknowledging it (22).

ECG adds conceptual value by integrating theoretical traditions that have previously remained separate. Grounded in Jodi Halpern's notion of “engaged curiosity,” and informed by narrative medicine and Foucault's medical gaze, ECG bridges scientific objectivity with emotional presence and offers a holistic alternative to the cognitive-affective dichotomy (22). The ECGS provides the practical value of measuring this curiosity-driven, emotionally attuned perceptual stance. Existing instruments such as the JSE, CARE, and TES assess cognitive perspective-taking or relational behaviors but do not capture the perceptual orientation that guides them (22). The ECGS therefore offers a new operationalization of an aspect of clinical empathy that remains unaddressed in current tools.

Emotional intelligence (EI) has been increasingly recognized as a foundational competency in healthcare, supporting patient centered communication, teamwork, and clinical decision making (23). Classic EI frameworks emphasize distinct cognitive and behavioral abilities such as emotion recognition, regulation, and interpersonal effectiveness, which directly facilitate a clinician's capacity to build meaningful patient connections (24). Social competence similarly encompasses these relational skills, confirming that interpersonal sensitivity and collaborative communication are critical precursors to effective, therapeutic patient encounters (25). While empathy is often treated as a core component of EI, EI frameworks typically emphasize the cognitive dimension of understanding others' emotions. The ECG extends this by integrating emotional attunement, cognitive understanding, and engaged curiosity into a unified perceptual stance oriented toward clinical reasoning and patient care. In this sense, ECG can be viewed as a clinically specific extension of EI, because it draws on foundational EI abilities such as emotion recognition and regulation, yet highlights dimensions often underrepresented in EI training, particularly curiosity-driven emotional engagement. At the same time, ECG remains conceptually distinct because it frames empathy not as a discrete skill, but as a unified perceptual stance grounded in engaged curiosity that shapes how clinicians interpret and respond to patients' emotional worlds. Thus, ECG both overlaps with and extends EI, offering a clinically grounded refinement of empathy within the broader landscape of social competencies.

While empathy is often treated as a core component of emotional intelligence (EI), EI frameworks typically emphasize the cognitive dimension of understanding others' emotions. The ECG extends this by integrating emotional attunement and engaged curiosity, thereby reframing empathy as a unified perceptual stance. In this sense, ECG can be viewed as a clinically specific manifestation of EI, but one that highlights dimensions which are often underrepresented in EI training. EI may provide the foundational skills necessary to sustain ECG, such as emotion recognition and regulation, yet ECG also contributes back to EI by cultivating curiosity driven emotional engagement in patient encounters. Thus, ECG both overlaps with and extends EI, offering a clinically grounded refinement of empathy within the broader landscape of social competencies.

Building on Halawi et al. (22) previous work, the present study extends the analysis of the original dataset by examining the levels of an empathetic clinical gaze among Swedish medical students using the ECGS and exploring how these levels vary across demographic groups. Although the present study uses the same participant sample and ECGS assessments as the initial psychometric validation study (22), it addresses distinct research questions. Whereas the previous publication focused on scale development, content validation, internal consistency reliability, and exploratory factor analysis, the present study investigates demographic variation in ECGS scores, incorporates previously unanalyzed open-ended written comments, and provides a theoretically informed interpretation of the findings using Bourdieu's Theory of Practice. Therefore, the scientific contribution of the present study lies both in extending the validation of the ECGS through demographic analyses and in demonstrating how clinical empathy is socially shaped within medical education using Bourdieu's Theory of Practice.

Accordingly, this study aimed to examine the following research questions (RQ):

- RQ 1: Differences in empathetic clinical gaze levels among Swedish medical students using the ECGS.
- RQ 2: How demographic characteristics relate to empathetic clinical gaze levels.
- RQ 3: Students' perceptions of an empathetic clinical gaze within educational and clinical contexts, interpreted through Bourdieu's Theory of Practice.

### Theoretical framework

Bourdieu's Theory of Practice encompasses three key concepts: habitus, capital, and field, which illustrate the interaction between social structures (the external world) and individual behaviors (the inner world) (26–28). In medical education, the inner world represents medical students and their professional norms, while the outer world pertains to their educational and clinical settings. The perspectives and behaviors of students and the social structures they encounter mutually influence one another (28). According to Bourdieu, social practices stem from an individual's dispositions (habitus) and accumulated power (capital) within a social field (26–28). Therefore, medical students' actions regarding empathy are shaped by the available capital in their educational and clinical environments and their habitus. This study aims to explore the practice of empathy through the interplay of habitus, capital, and field by inductively analyzing the gathered data in relation to Bourdieu's theory.

## Materials and methods

### Study design

A cross-sectional quantitative survey with a secondary qualitative content analysis of open-ended responses was carried out (29). The open-ended questions were originally developed for instrument item evaluation, however, the depth of the spontaneous participant responses regarding clinical empathy prompted a *post-hoc* qualitative analysis, which prompted the addition of a *post-hoc* research question (RQ3) to explore emergent themes. Qualitative comments were linked directly to participants' demographic metadata such as academic year within the survey platform to ensure rigorous analytic integration.

### Relation to previous publication

The quantitative participant sample (*n* = 251) and ECGS measurements used in this study are the same as those reported in the original psychometric validation of the ECGS (22). That previous publication focused exclusively on instrument development, content validation, internal consistency reliability, exploratory factor analysis, and initial psychometric performance. The present study addresses different research questions and therefore represents a secondary analysis of the same dataset. Specifically, it examines demographic differences in ECGS scores that were not previously reported, analyzes the brief to moderately reflective open-ended written comments that were not included in the validation study, integrates quantitative and qualitative findings, and interprets these findings using Bourdieu's Theory of Practice. None of the qualitative analyses, integrated interpretation of quantitative and qualitative findings, theoretical framing, or discussion presented here appeared in the previous publication (Supplementary material 1).

### Sample & participants

A convenience sampling strategy was used to recruit medical students in Sweden through closed Facebook groups associated with Swedish medical programs. Eligible participants were required to be currently enrolled in a Swedish medical program, at any year of study, to have completed at least one clinical placement, and to identify as male or female. Because all Swedish medical programs are offered at public universities, the sample consisted exclusively of students from public institutions. To ensure that respondents were genuine medical students, recruitment was conducted through closed Facebook groups moderated by university-affiliated students acting as administrators of the groups, where membership is restricted to enrolled students. One of the researchers (LH) requested access to each group in order to post the study invitation, ensuring that only verified medical students could view and respond to the survey.

The survey invitation was posted in semester-specific groups at Göteborgs universitet, Örebro universitet, and Karolinska Institutet, as well as in larger university-wide groups (Uppsala ~3.7k members, Lund ~2.9k members, Umeå ~2.6k members, Linköping ~1.5k members) and national groups (“Läkarstudenter i Sverige” ~5.7k members, “KI läkarstudenter” ~3.7k members). Because many students might have belonged to multiple groups, for example, both their semester group and one or more national groups, there might have been substantial overlap between memberships. As a result, the number of students potentially exposed to the invitation was considerably lower than the sum of all group members. Based on group sizes and expected overlap, it was estimated that approximately 500–1,000 students may have been exposed to the invitation. The exact number of students who viewed or accessed the survey link could not be determined, as Google Forms does not track link views.

A total of 255 students consented to participate; however, four students who did not meet the criteria were excluded and data from 251 participants remained for analysis. Of these, 50 students provided qualitative responses to open-ended questions regarding the newly developed empathetic medical gaze scale items. Although the responses varied in length, from brief clarifications to short reflective remarks, they offered analytically meaningful insights that were included in the qualitative analysis.

### Instrument: empathetic clinical gaze scale

*Demographic variables:* Demographic variables were age, gender, academic year (1–3 & 4–6), and clinical experience (yes, no).

The ECG-scale (22) was used to assess the level of empathetic medical gaze among medical students. The ECGS was developed directly from the theoretical framework of the ECG, which conceptualizes clinical empathy as a single, integrated perceptual stance rather than a set of separate components. It assesses how cognitive understanding and emotional attunement manifest in medical school students' perceptual attitudes (22). Guided by this definition, the item-development process focused on translating ECG's core features; cognitive understanding and emotional attunement, into observable attitudes and beliefs that together reflect one unified construct. Cognitive understanding is captured in items that address the clinician's effort to interpret the patient's narrative, concerns, and broader life context. Emotional attunement is reflected in items concerning attention to non-verbal cues, sensitivity to how emotions shape clinical presentations, and openness to patients' affective expressions (22). These facets are not treated as distinct dimensions or subscales; instead, they represent interdependent expressions of the same empathetic clinical gaze. In this way, the ECGS operationalizes ECG as a holistic construct in which cognitive and affective elements are inherently intertwined (22).

The ECG-scale consists of 16 items on a Likert-type scale ranging from “1” indicating (strongly disagree) to “4” indicating (strongly agree), focusing on a single domain, namely, “engaged curiosity,” i.e., a perceptual attitude that configures itself as a genuine interest toward patients through emotional attunement. The scale items capture two overarching domains (22):

***(1) Perceived importance of patient emotions and perspectives***, i.e., the extent to which medical students believe patients' feelings, non-verbal cues, and subjective experiences influence diagnosis, treatment, and outcomes.

***(2) Perceived clinical value of empathy***, i.e., the extent to which empathy is viewed as a contributor to diagnostic accuracy, therapeutic effectiveness, patient outcomes, and overall clinical competence.

The total score of the scale ranges from 16 to 64, with a higher score (≥56) indicating higher empathetic medical gaze (22). Initial testing of the psychometric properties of the ECG scale resulted in good reliability with a Cronbach's alpha of 0.80 among medical students and corrected item-total correlations (0.37–0.60) (22). The ECG-scale was transformed into an online questionnaire via Google Forms. The survey included two open-ended questions at the end of the instrument:

***(1) “Which statement(s) were unclear, if any?”***

***(2) “Which statement(s) were irrelevant, if any?”***

These prompts were included as item-evaluation questions about clarity and relevance rather than as interview-style questions designed to elicit in-depth narratives. Accordingly, the written responses, ranging from brief clarifications to more reflective remarks, are treated as concise qualitative comments embedded within a survey instrument.

### Data collection and ethical considerations

The study, conducted in 2023, did not require formal approval from the Swedish Ethical Review Authority but was approved by the Health Sciences Ethics Council at Kristianstad University (Dnr: U2022-2.1.12-2110). It followed the recommendations of the Helsinki Declaration for research involving human subjects (30).

The process for data collection involved individual Facebook posts that included a link to a questionnaire, along with brief information about the study shared in Facebook groups connected to medical students at seven Swedish universities. Participants were directed at a comprehensive description of the study and a consent statement. After giving their consent, they accessed the online questionnaire, which featured open-ended questions at the end for them to elaborate on the topic of empathy. Data collection took place from March 14, 2023, to August 1, 2023.

### Data analysis

#### Quantitative analysis

The analysis began by assessing the dependent variable's (total ECG score) distribution, which showed a non-normal distribution using Kolmogorov-Smirnov and Shapiro-Wilk tests. The study proceeded with a descriptive analysis to outline the demographic traits of the participants using frequencies, means, and standard deviations where appropriate. This was followed by an inferential analysis phase that utilized the Mann-Whitney *U*-test for group comparisons. Level of significance was set at *p* < 0.05.

#### Qualitative analysis

The qualitative analysis was conducted using inductive qualitative content analysis following the methodology laid out by Graneheim and Lundman (31), complemented by a later interpretive application of Bourdieu's Theory of Practice. After translating the students' open-ended responses from Swedish to English, the material was read several times to gain a sense of the whole. During this process, meaning-bearing units were identified. In this study, a meaning-bearing unit was any part of a student's response that expressed an experience, perception, or reflection related to empathy in educational or clinical settings. These meaning-bearing units were then condensed, while preserving its core meaning. Each condensed unit was then assigned a code, which is a short descriptive label that captures the essence of what the student expressed. The codes were then compared for similarities and differences and grouped into subthemes based on whether they expressed similar ideas or underlying meanings, even if they used different wording. Grouping was therefore based on conceptual similarity and relevance to the research question (RQ 3), not on frequency. Less common but conceptually important insights were retained when they contributed meaningfully to understanding students' perceptions of an empathetic clinical gaze. The subthemes were subsequently abstracted into overarching themes that captured the latent content, i.e., the deeper, underlying meaning across students' reflections. These themes represented broader patterns in how students described the learning, enactment, and challenges of empathy in clinical and educational contexts.

Importantly, the development of meaning-bearing units, codes, subthemes, and themes was conducted inductively, without applying Bourdieu's theory during the coding process. Only after the inductive themes had been fully established was Bourdieu's Theory of Practice applied in a separate interpretive step. This theoretical lens helped illuminate how habitus, capital, and field shaped students' descriptions of empathy and how these social structures influenced their development of an empathetic clinical gaze. Thus, theory informed the interpretation of the themes, not their construction.

To ensure credibility and trustworthiness, all themes and supporting quotations were reviewed collaboratively by the authors (LH, AK), and consensus was reached on the final thematic structure and representative citations.

### Integration of quantitative and qualitative findings

Research Question 3 (RQ3) was introduced *post-hoc* following the emergence of brief to more reflective qualitative comments on clinical empathy within the open-ended responses on item evaluation. The qualitative dataset comprised 50 written comments linked to participants' demographic metadata (e.g., academic year) within the survey platform, enabling analytic integration. This linkage allowed contextualization of quantitative patterns, generating developmental insights that neither dataset alone could reveal.

## Results

### Overview of results

Quantitative results showed moderate ECG levels overall, with significant differences between academic years but not between genders or age groups. Qualitative content analysis generated two themes describing how students understand and apply empathy in clinical settings. Interpreted together, the results convey how institutional norms and clinical socialization shape students' emotional engagement and may help contextualize the differences observed between academic year groups.

This study examined empathic clinical gaze (ECG) among Swedish medical students using a cross-sectional quantitative survey supplemented by a secondary qualitative content analysis of brief to moderately reflective written comments.”

### Quantitative results

The majority of the 251 participating students were females (78%) and aged between 18 to 24 years (59%). Students from all years of medical studies were represented, with a notable concentration in the fifth year (Table 1). The mean score of empathetic clinical gaze for the whole sample was 53.4 (SD = 6.2), indicative of relatively “average” scores. Concerning the levels of ECG scale scores, no significant differences were found between males and females (*p*-value = 0.128) or age groups (*p*-value = 0.790). However, a significant difference was found between academic years (*p*-value = 0.030), with year 1–3 students having higher ECG than students in year 4–6 (Table 1).

**Table 1:** Comparison of groups with high, average, and low empathetic clinical gaze levels based on participants' sociodemographic characteristics.

Characteristics	*N*	ECG, *n* (%)			*p*-value^*^
		High, *n* = 83	Average, *n* = 89	Low, *n* = 79	
Gender					0.128
Male	56	12 (21.4%)	23 (41.1%)	21 (37.5%)	
Female	195	67 (34.4%)	66 (33.8%)	62 (31.8)	
Age					0.790
18–24 years	148	47 (31.8%)	50 (33.8%)	51 (34.5%)	
≥25 years	103	32 (31.1%)	39 (37.9%)	32 (31.1%)	
Medical school year					0.030
Year 1–3	110	43 (39.1%)	36 (32.7%)	31 (28.2%)	
Year 4–6	141	36 (25.5%)	53 (37.6%)	52 (36.9%)	

Data is presented as *n* (%) in rows. ^*^Results of Mann Whitney U analysis.

### Qualitative results

The content analysis brought forth two main themes:(1) Clinical *Detachment- The practice of applying empathy in a clinical context* and (2) *The application and significance of empathy in patient encounters* (Table 2).

**Table 2:** An overview of sub-themes and themes development.

Sub themes	Main themes
*Learned Behavior*	*Clinical detachment- The practice of applying empathy in a clinical context*
*Obstructing factors in the facilitation of empathy*	
*Empathy's importance in diagnosis establishment*	*The significance of empathy in patient encounters*
*Appropriate level of empathetic engagement*	

#### Theme 1: clinical detachment- the practice of applying empathy in a clinical context

The first theme, derived from the subthemes *learned behavior* and *obstructing factors*, highlights the relationship between clinical detachment and empathy among medical students. While students acknowledge the importance of empathy in medical practice, they also advocate for a cognitive rather than affective approach, suggesting that physicians need to distance themselves from patients' emotions to cope, a behavior developed through experience.

As one student expressed, emotional distancing becomes a learned way of managing the emotional load of clinical work:

“*Disconnecting empathy more or less temporarily is a type of coping strategy that you learn as a doctor.” (Respondent 54, male, year 3)*

The pursuit of clinical detachment through emotional distancing among students is seen as essential for aspiring physicians to protect themselves from emotional exhaustion. This behavior helps them avoid becoming overly involved in patients' feelings, which can negatively impact their ability to cope with the demands of the medical profession, and the distress patients experience.

One student described the emotional burden of patient suffering:

“*Too much empathy, you will not be able to cope with all the suffering around you.” (Respondent, 127, male, year 4)*

The students argued that practicing empathy is complex in a clinical context, suggesting that various factors can hinder empathetic expression and lead to a sense of clinical detachment, with one student expressing:

“*As with everything there is a gray scale. Sometimes there isn't time or energy to get into a patient's emotional life and it doesn't have to be negative for the patient in question.” (Respondent 190, female, year 2)*

Medical practice, based on student responses, appeared to be influenced by institutional forces that promote empathetic expression while encouraging clinical detachment. Key challenges identified include time constraints, burnout, heavy workloads, and a focus on scientific objectivity. The heavy workload associated with managing numerous patients makes it difficult for physicians to fully engage with patients' thoughts and feelings, resulting in a perceived link between practicing empathy and increased burnout, with one student expressing:

“*I think it makes high demands if you always put yourself in the perspective of others, considering how many patients doctors see.” (Respondent 231, female, year 5)*

Furthermore, it was emphasized that the significance of the scientific clinical gaze in physician-patient interactions, highlighting the crucial role of scientific medical knowledge in competence and clinical judgment, particularly in diagnostics. It asserts that the scientific gaze is a primary tool in a clinical context, which aligns with one student's expression:

“*The most important feature is the doctor's clinical gaze. You don't have time to empathize with a patient's pain or losses, for example, if you are to manage to arrive at a diagnosis quickly or even cope with a professional life.” (Respondent 224, female, year 5)*

Overall, the medical students equate affective empathy with burnout, emphasizing scientific objectivity and the need for clinical detachment. They advocate for impersonal care due to time constraints in diagnostics and the demands of medical professionalism, viewing empathy as time-consuming and draining. However, as one student (respondent 127) noted that a lack of understanding of the person behind the disease can hinder diagnostics, another (respondent 190) argued that insufficient time or energy to empathize doesn't necessarily harm the patient. These highlight differing views on empathy, particularly the separation of cognitive and affective empathy in understanding patients with students tending to prioritize cognitive empathy, or clinical detachment, over affective empathy due to various obstructing factors.

#### Theme 2: the application and significance of empathy in patient encounters

This second theme was derived from two sub-themes as well: *Empathy's importance in diagnosis establishment* and a*ppropriate level of empathetic engagement*.

Students believe that understanding a patient's emotional perspective is not always essential for swift diagnosis; it's considered circumstantial. They generally feel that empathy does not significantly affect diagnostic accuracy, especially for somatic conditions like a broken leg or hip, where patient feelings are deemed irrelevant to the diagnosis. However, empathy is seen as more relevant for psychological conditions. One student noted that the importance of empathy in diagnosis varies based on the specific situation, highlighting different encounters such as health habits versus surgical procedures:

“*Personally, I believe that empathy can be important in the diagnosis of psychological illnesses and in the choice of treatment for somatic illnesses, but not in the diagnosis of somatic illnesses. It could be worthwhile to differentiate between these in the questions. Asking about the latter, i.e., diagnosis and feelings when it only concerns somatics, I think feels irrelevant.” (Respondent 54, male, year 3)*

One student emphasized the importance of empathy in diagnosis, noting that its relevance varies with the type of patient and their situation. They explained that the significance of empathy is disease dependent. Another student pointed out that while understanding a patient's context is crucial for taking medical histories and planning follow-ups, it may not impact surgical outcomes as long as the surgeon has the necessary skills:

“*Responding empathetically is a balancing act, as different occasions require different responses. But specific cases and conditions are required for more adequate answers. Some diagnoses do not require empathic ability to treat and diagnose, other diagnoses require good empathic ability for the most favorable outcome possible”. (Respondent 48, female, year 2)*

Students perceive the emotional perspective of patients as lacking scientific value, particularly in diagnostics, viewing emotions as unimportant for clinical judgments. While they recognize emotional vulnerabilities and the experience of empathy, they consider these emotions burdensome for both themselves and patients. Empathy is seen as circumstantial, depending on illness, patient type, and physician's role, leading to challenges in appropriately expressing empathetic engagement in patient interactions.

“*There is a difference in understanding someone else's feelings, and actually being able to convey it, i.e., empathy as a communicative act that is perceived by the recipient.” (Respondent, 202, female, year 2)*

The importance of empathy in medicine is widely recognized by the students, but achieving effective empathetic interaction between physicians and patients is said to be challenging due to differing interpretations of similar words and actions. This issue is particularly notable when delivering bad news, which can be painful for patients and lead to denial, complicating diagnoses. Additionally, demonstrating understanding of a patient can be difficult; physicians may focus on technical aspects of care and fail to convey empathy, thus hindering the practical application of their understanding, with one student expressing:

“*It is important for the doctor to be empathetic, but sometimes the doctor may have to say things in the moment that hurt or become difficult for the patient, and too much empathy or displeasure in the face of the patient's reluctance can lead to poorer diagnostics. This may apply to a patient who does not want to collect information about their health condition”. (Respondent, 207, female, year 3)*

### The integration of quantitative and qualitative results

Interpreted together, the quantitative ECGS scores and the qualitative written comments suggest how institutional norms and clinical socialization shape students' emotional engagement. The quantitative analysis revealed no statistically significant gender-based differences in ECG levels (*p* = 0.128), contrasting with variations observed across medical school years (*p* = 0.030), where early-year students demonstrated higher ECG levels (39.1% high ECG in years 1–3 vs. 25.5% in years 4–6). Qualitative findings contextualize these patterns, with themes such as clinical detachment as a learned behavior and empathy's role in diagnostic accuracy suggesting that institutional training norms and clinical socialization processes may homogenize gender perspectives, while helping to contextualize why later-year students scored lower than earlier-year students. For instance, students described empathy as a circumstantial skill shaped by workload pressures and the need for scientific objectivity, factors that align with the lower ECG scores observed among later-year students. Simultaneously, shared narratives about empathy as a coping strategy and its ascribed relevance in patient encounters may explain the lack of gender divergence, as both male and female students emphasized balancing emotional engagement with professional detachment. This integration highlights how structural and experiential factors, rather than demographics, may help contextualize differences in ECGS scores across academic years in Swedish medical education.

## Discussion

This study explored the perception of empathetic medical gaze (ECG) among medical students in Sweden. It found no significant difference in ECG between female and male students or age groups, although early-year students exhibited higher ECG levels than those in later years. The lack of significant gender or age differences in ECG among Swedish medical students challenges traditional assumptions about empathy's determinants. While some research suggests women demonstrate higher cognitive empathy due to socialized roles or biological factors (32), the similar ECG levels may result from standardized training norms emphasizing patient-centered communication (33, 34). The absence of age-related variation contrasts with findings that indicate declines in cognitive empathy with age (35) and may be related to the narrower age range studied (18–24 vs. ≥25). Furthermore, the lower ECGS scores observed among later-year students parallels global trends linking clinical immersion with reduced empathy, often due to burnout and workload pressures (33, 36). Longitudinal studies from Italy and Iran also report decreases in empathy scores during clinical training, with factors like “scientific objectivity” and “time constraints” diminishing compassionate care (33, 34). Taken together, these findings provide new insights from a Scandinavian context, demonstrating that structural factors such as curriculum design, rather than demographic characteristics, play a central role in shaping ECG. This underscores the need for educational interventions to uphold empathy in clinical settings (36).

The qualitative written responses suggest that students conceptualize empathy as both essential and burdensome; however, because these comments were elicited through item-evaluation prompts, they should be interpreted as brief to moderately reflective and hypothesis-generating rather than as equivalent to interview- or focus-group evidence. Students described clinical distancing as a learned coping strategy, emphasizing cognitive empathy while effectively distancing themselves to manage emotional exhaustion. Time pressure, heavy workload, and the dominance of the scientific gaze were perceived as barriers to empathic engagement. These barriers closely mirror findings from a recent qualitative synthesis showing that levels of empathy are frequently associated with time constraints, prioritization of biomedical knowledge, institutional culture, and pressures arising during clinical training (37). Although students recognized the importance of empathy in patient interactions, they also noted that its relevance varied depending on the diagnosis, clinical context, and communication demands. Empathy was described as situational, strained, and sometimes at odds with the perceived need for diagnostic efficacy. These findings are consistent with recent mixed-methods studies reporting that medical students often recognize empathy as clinically important while simultaneously experiencing tensions between emotional engagement and professional demands (17–19). Communication-skills training studies have shown that students distinguish between understanding patient emotions and effectively expressing empathy in practice, whereas experiential interventions such as improvisation training and virtual-reality simulations have highlighted the importance of reflection and emotional awareness in sustaining empathic engagement.

**Figure 1:**
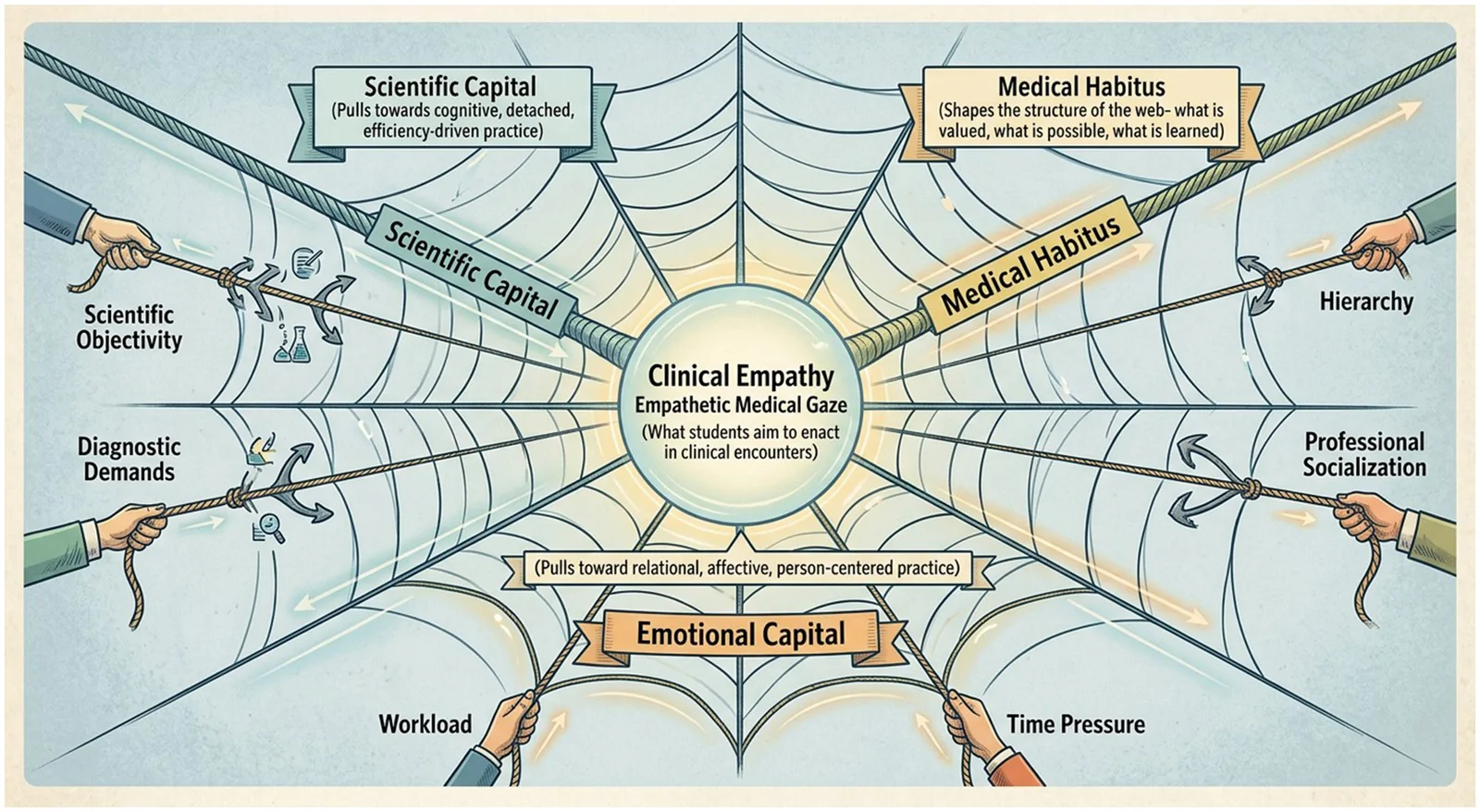
A spider-web conceptual model illustrating how clinical empathy is shaped, stretched, and constrained within medical education. At the center is clinical empathy, i.e., the empathetic clinical gaze that students strive to enact in clinical encounters. The surrounding strands represent scientific capital, emotional capital, and medical habitus, each exerting tension on the center. External field pressures are grouped according to the strand they most strongly influence: scientific objectivity and diagnostic demands (pressures toward detached, efficiency-driven reasoning within scientific capital); workload and time pressure (constraints on relational and affective engagement within emotional capital); and hierarchy and professional socialization (norms and expectations learned through medical habitus). Together, these forces visualize the dynamic negotiation between cognitive and affective empathy identified in the qualitative findings, showing how students' empathetic practice is continuously shaped by structural conditions in medical training. The figure was created using Adobe Firefly (free version, Adobe Systems).

In addition, these patterns can be understood through Bourdieu's theory of practice, particularly the concepts of habitus, field, and capital (26–28). In medical students, clinical detachment reflects their habitus and influences their relationships with patients, aligning with Bourdieu's theory of practice that emphasizes social fields such as educational and clinical settings, where exchanges occur (26–28). These fields have specific rules and traditions that impact a person's social standing. The medical field's educational and clinical environments are spaces where medical students navigate their social positions to become physicians, often prioritizing scientific medicine over its art, leading to clinical detachment. Bourdieu posits that various forms of capital are essential for social standing within any field, with valued resources seen as capital. Students' descriptions of emotional restraint and cognitive distancing reflect the early formation of a professional habitus shaped by the norms of the medical field. In this field, scientific capital, which exhibits itself as biomedical knowledge, diagnostic reasoning, and efficiency, is highly valued, while emotional capital is recognized but often constrained by institutional expectations. Similar findings have been reported, reinforcing the idea that empathy is shaped by broader educational and cultural experiences rather than being a fixed personal characteristic (38), or individual dispositions (33). As students navigate educational and clinical environments, they learn which dispositions are rewarded and gradually internalize clinical distancing as part of their professional identity. Figure 1 illustrates this dynamic and shows how empathy is negotiated within the interplay of habitus, capital, and field.

These findings align with research on EI and social competencies in healthcare. EI mainly focuses on cognitive empathy, i.e., understanding others' emotions, rather than affective empathy (25). This aligns closely with how students in our study described empathy, namely as something that must be controlled, regulated, and used selectively to avoid emotional exhaustion. Students repeatedly emphasized coping strategies, emotional distancing, and the need to stay efficient under time pressure and heavy workload. These are all forms of emotion regulation, which are central components of EI. EI training has been shown to improve communication and stress management (39), and this mirrors students' accounts of balancing empathy with diagnostic demands and using cognitive empathy to remain composed in challenging encounters. The ECG can therefore be understood as a clinically specific extension of EI because it uses core EI abilities, such as emotion recognition, regulation, and cognitive understanding, as its foundation. However, ECG is also distinct from EI, because instead of treating empathy as a single skill, ECG describes a broader perceptual stance that involves engaged curiosity and affective attunement toward the patient. This distinction helps explain why later-year students scored lower on ECG. EI-related skills alone may not sustain emotional engagement when students face structural pressures such as workload, time constraints, and the dominance of the scientific gaze. ECG therefore highlights aspects of emotional engagement that EI frameworks do not fully capture, pointing to the need for learning environments that support curiosity-driven and relational forms of empathy alongside cognitive and regulatory skills.

What makes these findings important is that they show empathy not as an individual trait that students “misplace,” but as a socially conditioned practice shaped by the structural logic of medical education. The differences in ECGS scores across academic years appear less to be a personal deficit and more a predictable adaptation to the demands of the clinical field, where efficiency and scientific reasoning are privileged. This reframes differences in empathy-related perceptions as a systemic problem rather than a student-level failure, especially since changes in empathy might be less pronounced than commonly assumed and shaped by contextual influences during training (40). The implications for medical education are significant. If empathy is shaped by field norms and professional habitus rather than by gender or age, interventions must target the learning environment rather than individual students. This can include designing syllabuses that explicitly value emotional capital alongside scientific capital, by integrating reflective practice and supervision during clinical involvement, and managing workload and time pressures that limit empathetic engagement, by supporting students in developing sustainable forms of empathy that do not lead to emotional exhaustion. Recent evidence also suggests that community-based and service-learning approaches may help sustain empathy throughout professional development by promoting meaningful engagement with patients and communities (41).

The integration of quantitative and qualitative data reveals a compelling cross-sectional alignment in empathy-related perceptions across academic years. Quantitatively, a significant drop in ECGS scores was observed between early-year and later-year students (*p* = 0.030). The qualitative findings offer a plausible contextual framework for interpreting this shift, reflecting a shared narrative around professional socialization. Specifically, students in years 2 and 3 frequently identified “*disconnecting empathy”* as an active coping strategy to protect against burnout. In later years, this strategy was described as a response to systemic workplace constraints, as illustrated by a year 5 student. Rather than representing isolated feedback, these spontaneous open-ended remarks illuminate potential underlying mechanisms that may help conceptualize and operationalize the statistical differences in empathy scores observed among the senior student groups.

Ultimately, this study contributes a theoretically informed understanding of how empathy may be shaped in medical education, sheds light on factors that may help explain differences in empathy-related perceptions across academic years during clinical training and identifies structural levers that educators can modify to support the development of an empathetic clinical gaze. This study imparts that empathetic practice among medical students is influenced by their habitus, with educational and clinical environments shaping interactions with patients. This often results in a preference for transactional interactions, as empathy is viewed as cognitive detachment rather than emotionally attuned, integrated interplay between cognitive and affective empathy (42). Such learned behaviors stem from the belief in traditional practices deemed successful, with detachment acting as a coping mechanism to avoid emotional exhaustion, reflecting medicine's historical focus on objectivity to preserve professional judgment (43). Hence, past experiences shape the empathy developed in medical education, demonstrating how habitus influences actions based on expected outcomes (26–28). However, not all studies have observed empathy erosion. A recent longitudinal study conducted in Spain reported significant increases in empathy across six years of medical training following the implementation of structured reflective workshops and repeated empathy-focused educational activities, suggesting that curricular design may substantially influence empathy trajectories (44).

### Methodological limitations

The present study should be interpreted as a secondary analysis of an existing dataset rather than an extension of the ECGS validation itself. By combining quantitative group comparisons with previously unanalyzed qualitative comments and interpreting both through Bourdieu's sociological framework, the study provides explanatory insight into how empathy develops within medical education beyond the psychometric questions addressed in the original validation study.

The study's methodology has limitations that affect the findings. The use of social media for recruitment may have introduced selection bias, limiting sample diversity to those active on these platforms and excluding individuals with lower engagement or limited internet access, affecting the transferability of results to broader medical student populations in Sweden or other contexts (45). The lack of methodological triangulation also reduces transferability, as the complexity of the phenomenon may not have been fully captured (46). Reliance on open-ended questions allowed detailed responses but may have led to challenges in articulating complex thoughts, affecting the credibility of the findings (46). Lastly, qualitative content analysis is subjective and may be influenced by researcher bias; thus, researcher triangulation and a detailed audit trail were implemented to enhance confirmability (47). Furthermore, the qualitative component was constrained by the original purpose of the prompts, which were designed to evaluate item clarity and relevance rather than to generate in-depth qualitative accounts; therefore, the responses likely provide only partial and selectively prompted insight and should be interpreted as brief to more reflective qualitative survey comments (48, 49).

Given the cross-sectional design of this study, differences between early- and later-year students cannot be interpreted as developmental changes or evidence of reduced empathy. These patterns reflect group differences rather than within-individual change over time. Group effects and unmeasured contextual factors, such as curriculum structure, stress levels, or differences in clinical exposure, may also explain the observed variation in ECGS scores. Longitudinal research is therefore needed to determine whether an empathetic clinical gaze changes as individual students progress through medical education.

## Conclusion

This study highlights the role of medical professional habitus in promoting an empathetic clinical gaze with regards to patient care among medical students. Findings indicate that the clinical detachment common in medical practice can lead to impersonal care, undermining the balance between cognitive and affective empathy, hence an empathetic clinical gaze. To address this, the study calls for reforms in medical education to better prepare students for an empathetic clinical gaze and suggests further research on medical students' experiences with clinical empathy in Sweden. A harmonious blend of clinical detachment and authentic empathy is vital for meeting patients' emotional needs, and emphasizing emotional attunement in medical training can improve physicians' responsiveness to patient concerns.

## Data Availability

The raw data supporting the conclusions of this article will be made available by the authors, without undue reservation.
