# Supplementary material for: Between scientific capital and emotional capital: a cross-sectional study on the empathetic clinical gaze in Swedish medical education
Source: Front Med (Lausanne). 2026 Aug 27;13:1876057. doi: 10.3389/fmed.2026.1876057 (PMC13558007; doi:10.3389/fmed.2026.1876057)
Supplement: Supplementary file 1 [file Table_1.docx]

Supplementary material X

Table 3. Relationship between the previous ECGS validation study and the present study.

| **Aspect** | **Previous publication** | **Present study** |
| --- | --- | --- |
| Participants | Same 251 medical students | Same participants |
| Dataset | Same quantitative dataset | Same quantitative dataset + previously unanalyzed qualitative comments |
| Primary aim | ECGS development and psychometric validation | Understanding empathy among medical students |
| ECGS scores | Reliability and validity | Demographic comparisons |
| Qualitative data | Not analyzed | Thematic analysis |
| Mixed-methods integration | No | Yes |
| Theoretical framework | No | Bourdieu's Theory of Practice |
| Principal contribution | Instrument validation | Explanatory understanding of empathy development |
